# A 4-Base-Pair Core-Enclosing Helix in Telomerase RNA Is Essential for Activity and for Binding to the Telomerase Reverse Transcriptase Catalytic Protein Subunit

**DOI:** 10.1128/MCB.00239-20

**Published:** 2020-11-20

**Authors:** Melissa A. Mefford, Evan P. Hass, David C. Zappulla

**Affiliations:** a Department of Biology, Johns Hopkins University, Baltimore, Maryland, USA; b Department of Biology and Chemistry, Morehead State University, Morehead, Kentucky, USA; c Department of Molecular, Cell, and Cancer Biology, University of Massachusetts Medical School, Worcester, Massachusetts, USA; d Department of Biological Sciences, Lehigh University, Bethlehem, Pennsylvania, USA

**Keywords:** RNA, RNP, TERT, TLC1, senescence, telomerase, telomerase RNA, telomere, two-hybrid screening, yeast

## Abstract

The telomerase ribonucleoprotein (RNP) counters the chromosome end replication problem, completing genome replication to prevent cellular senescence in yeast, humans, and most other eukaryotes. The telomerase RNP core enzyme is composed of a dedicated RNA subunit and a reverse transcriptase (telomerase reverse transcriptase [TERT]). Although the majority of the 1,157-nucleotide (nt) Saccharomyces cerevisiae telomerase RNA, TLC1, is rapidly evolving, the central catalytic core is largely conserved, containing the template, template-boundary helix, pseudoknot, and core-enclosing helix (CEH). Here, we show that 4 bp of core-enclosing helix is required for telomerase to be active *in vitro* and to maintain yeast telomeres *in vivo*, whereas the ΔCEH and 1- and 2-bp alleles do not support telomerase function. Using the CRISPR/nuclease-deactivated Cas9 (dCas9)-based CARRY (CRISPR-assisted RNA–RNA-binding protein [RBP] yeast) two-hybrid assay to assess binding of our CEH mutant RNAs to TERT, we find that the 4-bp CEH RNA binds to TERT but the shorter-CEH constructs do not, consistent with the telomerase activity and *in vivo* complementation results. Thus, the CEH is essential in yeast telomerase RNA because it is needed to bind TERT to form the core RNP enzyme. Although the 8 nt that form this 4-bp stem at the base of the CEH are nearly invariant among *Saccharomyces* species, our results with sequence-randomized and truncated-CEH helices suggest that this binding interaction with TERT is dictated more by secondary than by primary structure. In summary, we have mapped an essential binding site in telomerase RNA for TERT that is crucial to form the catalytic core of this biomedically important RNP enzyme.

## Abstract (precis)

The telomerase ribonucleoprotein (RNP) counters the chromosome end replication problem, completing genome replication to prevent cellular senescence in yeast, humans, and most other eukaryotes. The telomerase RNP core enzyme is composed of a dedicated RNA subunit and a reverse transcriptase (telomerase reverse transcriptase [TERT]). Although the majority of the 1,157-nucleotide (nt) Saccharomyces cerevisiae telomerase RNA, TLC1, is rapidly evolving, the central catalytic core is largely conserved, containing the template, template-boundary helix, pseudoknot, and core-enclosing helix (CEH).

## INTRODUCTION

Telomeres are repetitive sequences located at the ends of linear eukaryotic chromosomes. While they provide critical genome-protective functions, they are unable to be fully copied by DNA polymerases, due to the end replication problem. Short telomeres trigger a special G_2_/M cell cycle arrest known as senescence. In order to overcome the end replication problem and prevent senescence, most eukaryotic organisms require the ribonucleoprotein (RNP) enzyme complex telomerase (1).

The telomerase core enzyme consists of a dedicated noncoding RNA subunit (TLC1 in Saccharomyces cerevisiae) and a reverse transcriptase protein component (telomerase reverse transcriptase [TERT], or Est2 in S. cerevisiae). TERT utilizes a short template sequence in the telomerase RNA to iteratively add telomere repeats to the 3′ end of chromosomes (2). Together, these two core components are sufficient to reconstitute basal telomerase activity *in vitro* (3, 4). Telomerase RNAs are evolving strikingly fast, ranging in size from ∼150 nucleotides (nt) in ciliates to >2,000 nt in some species of yeast, with even the sequences and secondary structure models from closely related species within the same genus showing limited conservation. Experiments have shown that the 1,157-nt S. cerevisiae telomerase RNA, TLC1, exhibits a high degree of organizational flexibility. First, TLC1 acts as a flexible scaffold to bind the holoenzyme proteins in the RNP enzyme complex: i.e., the binding sites for Est1, Ku, and Sm_7_ can each be repositioned to novel locations within the RNA while supporting these subunits’ functions (5–8). Second, large portions of the RNA are dispensable for function *in vivo* and *in vitro* (3, 8–10).

Despite the wide array of differences between telomerase RNAs that have arisen during evolution, they do clearly share some key structural elements at their core (8, 11). These include (i) the template, (ii) the template-boundary element (TBE), (iii) a pseudoknot with base triples, (iv) a core-enclosing helix (CEH), and (v) an area of required connectivity (ARC).

In contrast to other conserved core elements, very little is known about the core-enclosing helix’s function. Being centrally located within the ARC, the CEH physically connects the pseudoknot to the template, enclosing the telomerase RNA’s core. However, when the CEH is deleted in the context of a functional circular permutation (i.e., maintaining RNA backbone integrity through the ARC), telomerase is inactivated *in vitro* (8), suggesting a key role for the core-enclosing helix beyond simply enclosing the core. Consistent with the CEH being essential, large deletions that encompass either the 5′ or 3′ side of the core-enclosing helix cause senescence and disrupt Est2 binding *in vivo* (10). Also suggesting that the CEH is a TERT-binding region, this area of TLC1 has been shown to interact physically with a protein via gPAR-CLIP (12) (an assay that assesses protein binding to RNA, but which does not identify the specific protein).

Here, we set out to investigate the structural and functional requirements of the core-enclosing helix in S. cerevisiae. We find that a core-enclosing helix of 4 bp is sufficient to provide telomerase function in yeast by promoting binding to TERT. There appears to be little sequence-specific requirement of the core-enclosing helix, indicating that TERT is generally binding double-stranded RNA at this region of TLC1 RNA. Together, these data convey the importance of the core-enclosing helix in yeast, while also helping to explain its changes in sequence during evolution.

## RESULTS

### The core-enclosing helix is required to prevent senescence and support telomerase activity.

**FIG 1:**
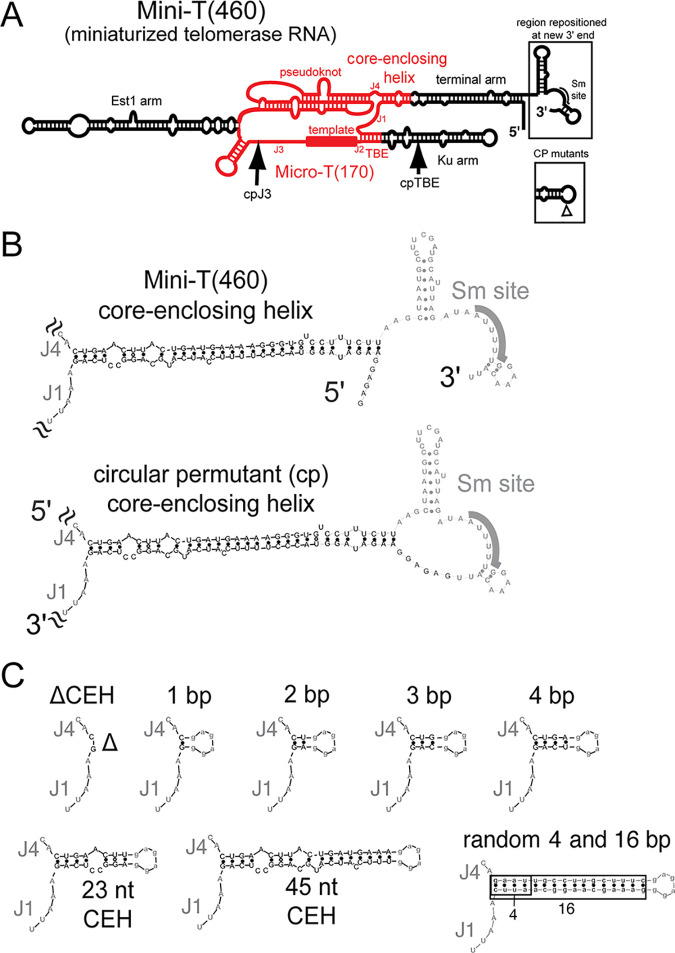
Core-enclosing helix mutations within circularly permuted Mini-T(460) yeast telomerase RNA. (A) The Mini-T RNA is a smaller yet still functional version of the 1,157-nt wild-type TLC1 RNA from S. cerevisiae (3). Shown in red is the Micro-T(170) RNA allele, which functions *in vitro* but not *in vivo*. The locations of the repositioned 5′ and 3′ ends of the circular permutations cpJ3 and cpTBE are indicated by arrows, and the boxes indicate changes to the terminal arm in these circular permutants. (B) Circular permutation of Mini-T was performed by connecting the 5′ and 3′ ends of the processed RNA forms (accomplished by circularly permuting the gene encoding the RNA). (C) Sequence of the core-enclosing helix mutants tested in Fig. 2 and 3.

Our previous results suggested that the core-enclosing helix (CEH) is required for activity *in vitro* in the context of a circularly permuted telomerase RNA allele comprising just the catalytic core, Micro-T(170) (8) (Fig. 1A). To investigate whether the core-enclosing helix is essential *in vivo* and to elucidate the structural requirements for function, we deleted or truncated the CEH in a circularly permuted larger telomerase RNA allele, Mini-T(460). Switching of the context from Micro-T to Mini-T is necessary since Micro-T lacks features necessary for telomerase function *in vivo*. Using a circularly permuted RNA is necessary to avoid disrupting the area of required connectivity (ARC) when studying the CEH, which is in the center of the ARC (8). Specifically, we chose the following two functional circular permutants: (i) cpJ3, which has the RNA ends between the template and the Est1 arm, and (ii) cpTBE, which has the ends in the Ku-binding arm upstream of the template-boundary element (8) (Fig. 1A and B).

**FIG 2:**
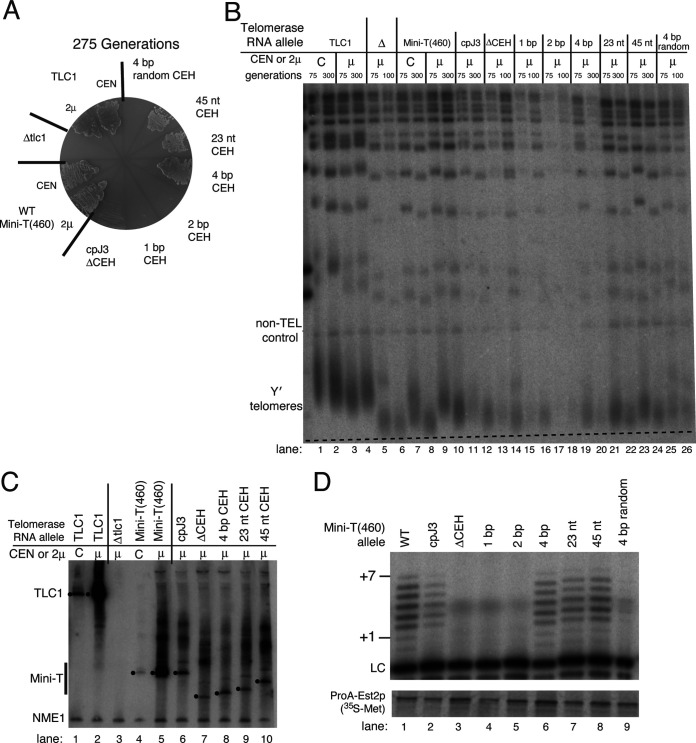
A minimal core-enclosing helix of 4 bp is sufficient for telomerase activity *in vitro* and *in vivo* in Mini-T(460) cpJ3. (A) Senescence assay. *RAD52*-null yeast strains containing only the indicated telomerase RNA allele were serially restreaked through 275 generations. All cpJ3 core-enclosing helix variants were expressed from high-copy-number 2μ plasmids. The cpJ3ΔCEH, 1-bp, and 2-bp mutants senesce, while the 4-bp and longer mutants support growth. (B) Telomere Southern blot. Viable cpJ3 core-enclosing helix mutants maintain short telomeres through 300 generations. Southern-blotted XhoI-digested genomic DNA was probed for telomeric repeat sequence and a chromosome IV (Ch. IV) control region. Note that the numbers of generations that yeasts were grown varied as indicated, depending on their viability in liquid culture. The dashed line represents the average length of Y′ telomeres in Mini-T(460) at 300 generations when expressed from a 2μ plasmid. The slant of the line reflects the migration of the Ch. IV control band. (C) Northern blot showing telomerase RNAs. Mature telomerase RNA is detectable from core-enclosing helix variants. Total cellular RNA was probed for either a fragment corresponding to Mini-T(460) or a 340-nt control RNA, NME1. The dots to the left of bands indicate the full-length RNAs, which differ in length according to the amount of core-enclosing helix sequence present. (D) Reconstituted telomerase activity assay. Only viable core-enclosing helix mutants show detectable *in vitro* telomerase activity. Radiolabeled products of telomerase reactions (+1 to +7) were separated on a 10% acrylamide denaturing gel by electrophoresis. A recovery and loading control (LC) was included in the telomerase reactions. Below, TERT protein containing [^35^S]methionine was separated by denaturing protein gel electrophoresis to show immunopurification of telomerase from the *in vitro* transcription and translation system.

We observed that the cpJ3 allele supported growth through 275 generations, as shown previously (8), whereas the cpJ3ΔCEH allele (Fig. 1C) senesced by 75 generations, similarly to a Δ*tlc1* strain (Fig. 2A). Senescing cpJ3ΔCEH cells exhibited telomeres that were shorter than those of the cpJ3 cells on telomere Southern blots (Fig. 2B, lanes 13 and 14). The inability of cpJ3ΔCEH cells to maintain telomeres did not seem to be due to RNA abundance, since we detected nearly the same level of ΔCEH RNA by Northern blotting as the functional cpJ3 allele (Fig. 2C, lane 7). Furthermore, we observed that reconstituted telomerase (3) using cpJ3ΔCEH was catalytically dead *in vitro* (Fig. 2D, lane 3). Thus, the core-enclosing helix is required for telomerase activity and function in cells.

**FIG 3:**
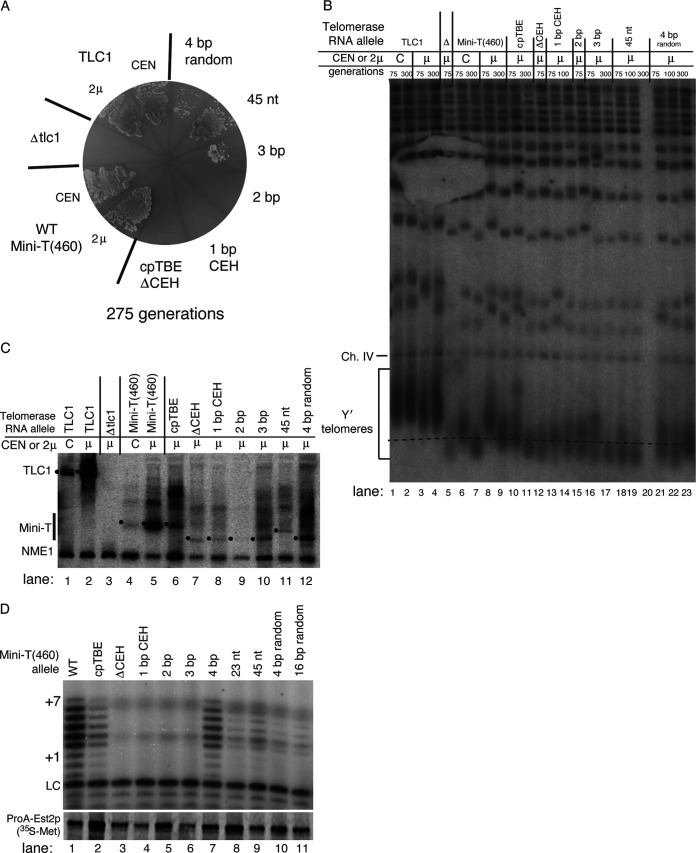
In Mini-T(460) cpTBE, a minimal core-enclosing helix of 3 bp prevents senescence and one of 4 bp supports robust enzyme activity *in vitro*. (A) Yeast strains expressing the Mini-T(460) 3-bp allele show stable small-colony growth on plates. Yeast cells were serially restreaked prior to the plate shown, as in Fig. 2. All core-enclosing helix variants were expressed from 2μ plasmids. (B) Viable core-enclosing helix mutants maintain telomeres, though they are shorter than wild-type Mini-T(460). Southern blotting was performed as for Fig. 2, with a dashed line aligned with the chromosome IV (Ch. IV) control band’s migration passing through wild-type 2μ Mini-T(460) at 300 generations. Some yeast cells could not be grown in liquid culture to the 300-generation time point, as indicated. (C) Mature telomerase RNA is detectable for all core-enclosing helix mutants. Northern blotting was performed as for Fig. 2. The location of the mature telomerase RNA is indicated by a dot to the left of each band predicted based on approximate size to be the processed form for each different TLC1 allele. (D) Telomerase activity *in vitro* is barely detectable for cpTBE with a CEH of 3 bp, while mutants with 4 bp or more of core-enclosing helix show detectable telomerase activity. *In vitro* telomerase activity assays were performed as for Fig. 2, with immunopurified Est2p below.

To further test the conclusion that the core-enclosing helix is essential in yeast telomerase, we examined telomerase function when the CEH was deleted in a different circular permutant, cpTBE. As in the context of cpJ3, we observed that cpTBEΔCEH senesced by 75 generations (Fig. 3A), concomitant with shortening telomeres observed by Southern blotting (Fig. 3B, lane 12). Senescence was again not due to lack of RNA accumulation, as the cpTBEΔCEH transcript was evident by Northern blotting at levels similar to those of other functional alleles (Fig. 3C, lane 7). Furthermore, the Mini-T(460) cpTBEΔCEH allele was catalytically dead *in vitro* (Fig. 3D, lane 3).

Together, the independent results from the contexts of both the cpJ3 and cpTBE circular permutations show that the core-enclosing helix is essential for telomerase function *in vivo* and RNP core enzyme activity *in vitro*.

### A core-enclosing helix of 4 bp is sufficient to support telomerase activity.

Given the functional necessity of the core-enclosing helix, we set out to determine what features of its structure are required. We began by adding back the native core-proximal base pairs one by one (Fig. 1C). This analysis revealed that just 4 bp of native core-enclosing helix sequence in cpJ3 supported telomerase function in preventing senescence (Fig. 2B) and telomerase activity with TERT *in vitro* (Fig. 2D, lane 6). A CEH of 3 bp in cpTBE caused an intermediate phenotype, barely allowing cells to avoid senescence (and causing slow growth of those that did survive) (Fig. 3A) and without clearly perceptible telomerase activity (Fig. 3D, lane 6). In contrast, 1- and 2-bp core-enclosing helices in cpJ3 or cpTBE did not support any telomerase function *in vitro* or *in vivo* (Fig. 2 and 3). For these alleles, it is likely that base pairing does not stably form. Thus, our data show that a minimum of 4 bp is required for substantial core-enclosing helix function.

Adding back more of the native core-enclosing helix also supported telomerase activity *in vivo* and *in vitro*, though with no detectable increases in activity over the shorter 4-bp helix (Fig. 2 and 3). Specifically, we added back either 23 nucleotides, which corresponds to the endogenous core-enclosing helix present in Micro-T(170), or 45 nucleotides. Both of these core-enclosing helices supported cell growth without evidence of senescence in the two circularly permuted Mini-T contexts (Fig. 2A and 3A), stably short telomeres (Fig. 2B, lanes 21 to 24, and Fig. 3B, lanes 18 to 20), and reconstituted telomerase activity (Fig. 2D, lanes 7 and 8, and Fig. 3D, lanes 8 and 9).

### The native core-proximal sequence of the core-enclosing helix is not necessary for function.

To determine whether the sequence of the native core-enclosing helix sequence is important, we tested nonnative base pairs. Interestingly, a sequence-randomized 4-bp helix (Fig. 1C [with 5′-side GAAU rather than CUGA in the wild type]) was able to support weak telomerase activity in the context of cpTBE, both *in vivo* (Fig. 3A and B, lanes 21 to 23) and *in vitro* (Fig. 3D, lane 10). The same random-sequence 4-bp helix in the context of cpJ3 was not sufficiently functional *in vivo* to prevent senescence (Fig. 2A), although it had weakly perceptible activity *in vitro* (Fig. 2D, lane 9). A longer 16-bp random helix (Fig. 1C) in cpTBE showed similar telomerase function to the 4-bp random helix (Fig. 3D, lane 11). These results suggested that the endogenous sequence of the core-enclosing helix has a noncritical, but also not nonexistent, role in its function.

**FIG 4:**
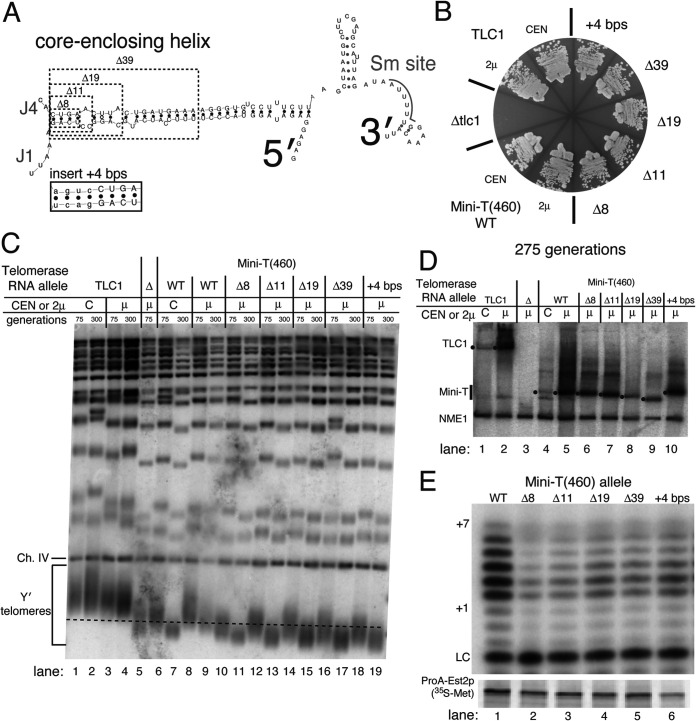
The core-proximal regions of the core-enclosing helix are dispensable for telomerase function *in vitro* and *in vivo*. (A) Schematic indicating the native core-enclosing helix nucleotide deletions tested. The inset shows the sequence of 4 bp inserted on the core-proximal side in the +4 bp allele. (B) Mutations that alter the length and nature of the core-proximal region of the core-enclosing helix prevent senescence in Mini-T(460). Yeast cells were restreaked on solid medium through 275 generations. All deletion or insertion mutants were expressed from high-copy-number 2μ plasmids. (C) Telomeres are shorter relative to Mini-T(460) when the core-enclosing helix is truncated or extended. Genomic DNA was Southern blotted as for Fig. 2. The dashed line represents the length of wild-type Mini-T(460) telomeres at 300 generations under 2μ expression levels for comparison. (D) Telomerase RNA is expressed from all mutants tested. Northern blots were performed as for Fig. 2, with dots indicating the position of the mature RNA in the gel. (E) Deletions and insertion cause slight defects in telomerase activity *in vitro*. Telomerase activity assays were completed as for Fig. 2. Immunopurified radiolabeled ProA-Est2 protein is shown below.

To further test the structural and functional requirements of the core-enclosing helix, we examined a series of deletions in non-circularly permuted Mini-T(460). If the particular sequence or the positioning of bulges in the core-enclosing helix is crucial for function, then deletion of these regions should abolish function. We first deleted the 4 bp nearest to the core (Δ8 in Fig. 4A). These are the same 4 bp that supported function in cpJ3 (Fig. 2). This Δ8 CEH truncation allele causes the core-enclosing helix to now begin at an asymmetrical 3-nt bulge, increasing the length of junctions J4 and J1 on either side of the helix, followed by 3 bp (Fig. 4A). This Δ8 deletion supported growth through 275 generations (Fig. 4B), although telomeres in these mutants were shorter than those of wild-type Mini-T(460) cells (Fig. 4C, lanes 8 and 9 versus lanes 10 and 11). The shorter telomeres in Mini-T(460)Δ8 do not appear to be due to lower RNA (Fig. 4D, lane 5 versus lane 6). However, the deletion of 8 nucleotides did partially decrease telomerase core enzyme activity *in vitro* (Fig. 4E, lane 1 versus lane 2).

Larger deletions of the core-enclosing helix also supported telomerase activity. We deleted 11 nucleotides [corresponding to the native nucleotides present in Micro-T(170)], 19 nucleotides, and 39 nucleotides (Fig. 4A). All of these deletions result in different core-enclosing helical structures near the core, yet each of these alleles supported telomerase activity *in vivo* and *in vitro* (Fig. 4B and E). The shortened telomeres (Fig. 4C) supported by these alleles do not appear to be due to insufficient levels of the RNAs (Fig. 4D).

Finally, we tested insertion of an additional 4 bp on the core-proximal end of the core-enclosing helix (Fig. 4A). This insertion (+4 bp) prevented senescence through 275 generations (Fig. 4B). Similar to the truncations of the CEH, the +4 bp insertion also caused telomere shortening relative to wild-type Mini-T(460) (Fig. 4C, lanes 18 and 19 versus lanes 8 and 9), not attributable to lowered telomerase RNA expression (Fig. 4D, lane 5 versus lane 10). The +4 bp insertion also supported telomerase activity *in vitro* (Fig. 4E, lane 6).

It is important to note that all 5 of these alleles (Δ8, Δ11, Δ19, Δ39, and +4 bp) generate a core-enclosing helix sequence abutting the catalytic core that differs from the wild type, yet all of these alleles function well both *in vivo* and *in vitro*. This further supports the conclusion that the native core-proximal sequence of the core-enclosing helix is not essential for telomerase function.

### The core-enclosing helix is required for telomerase RNA binding to TERT.

What is the mechanistic role of the core-enclosing helix in telomerase RNP function? Our results show that the CEH is required for both *in vivo* and *in vitro* telomerase action—thus, the CEH is essential for fundamental telomerase core enzyme activity. Since the core enzyme is composed of the TLC1 RNA and the TERT catalytic protein subunit, a parsimonious explanation for the contribution of CEH to enzyme function is that it is simply required for the RNA to bind to TERT to assemble the core enzyme.

**FIG 5:**
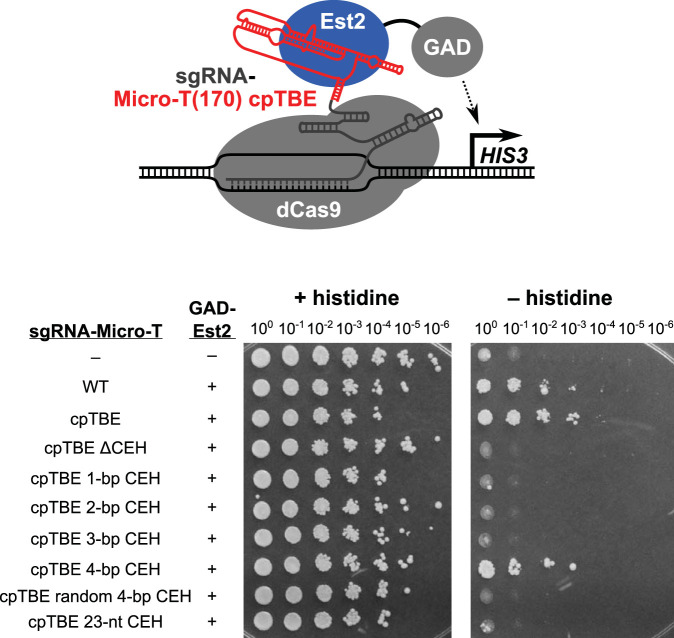
TERT-binding to telomerase RNA is impaired by a core-enclosing helix shorter than 4 bp. (A) Schematic of the CRISPR-assisted RNA-RBP yeast (CARRY) two-hybrid system (13). Different mutants of the TLC1 RNA core were fused to a CRISPR sgRNA, which was tethered to the promoter of a *HIS3* reporter gene by nuclease-deactivated Cas9 (dCas9). A fusion of Est2 protein to the Gal4 transcriptional activation domain (GAD) was also expressed, and activation of the *HIS3* reporter gene was used as a measure of binding between Est2 and the TLC1 RNA core. (B) A 4-bp core-enclosing helix is required for Micro-T(170) binding to TERT in the CARRY two-hybrid system. Expression of *HIS3* was assayed by growing cells to saturation in liquid culture, making six 10-fold serial dilutions, and applying these cells as spots to solid medium with or without histidine. In the GAD-Est2 column, “−” indicates expression of GAD that is not fused to Est2, while “+” indicates expression of the GAD-Est2 fusion protein. Similarly, “–” in the sgRNA–Micro-T column indicates expression of an sgRNA that is not fused to Micro-T.

To test the hypothesis that the CEH is a key binding site for TERT in telomerase RNA, we used the *in vivo* CRISPR-assisted RNA–RNA-binding protein (RBP) yeast (CARRY) two-hybrid RNA-protein interaction assay (13). This method employs catalytically inactive CRISPR/dCas9 (nuclease-deactivated Cas9) to tether an RNA of interest upstream of reporter genes in yeast such that if the RNA of interest binds to a protein (fused to a Gal4 transcriptional activation domain [GAD]), the RNA-protein binding interaction can be detected via *HIS3* reporter gene expression, allowing yeast to grow in the absence of histidine (Fig. 5A) (13). First, we tested if Micro-T(170) binds to TERT in the CARRY two-hybrid system. Indeed, the Micro-T(170) construct allowed significant growth on medium lacking histidine (∼1,000-fold greater than the negative control [Fig. 5B, second row]), indicating strong reporter gene activation based on binding to GAD-Est2 (TERT). Furthermore, circularly permuted Micro-T (cpTBE) also bound at least as well to GAD-TERT as unpermuted wild-type Micro-T (Fig. 5B, row 3). However, when we deleted the CEH from this cpTBE allele, there was a complete loss of TERT binding (Fig. 5B, compare rows 3 and 4). Micro-T cpTBE with a 1-, 2-, or 3-bp CEH did not bind TERT, but a 4-bp CEH did, consistent with performance of these alleles in telomerase function *in vivo* and *in vitro* (Fig. 2 and 3). We also tested binding by the random 4-bp and 23-nt CEH alleles, and these showed little or no *HIS3* activation. Given that these RNAs must be able to bind to TERT at least to some extent in order to support telomerase activity, presumably these alleles bind intermediately to TERT: i.e., too weakly for the CARRY two-hybrid system to detect (dissociation constant [*K_d_*] of ≥∼1 μM) (13), yet still sufficient to provide telomerase assembly and function, as shown in Fig. 2 and 3. Overall, we conclude that the CEH is required to bind to TERT and that 4 bp is sufficient for this critical interaction at the core of the telomerase RNP.

## DISCUSSION

A multisubunit enzyme cannot be active if its core components do not assemble. Despite this basic criterion for function, relatively little is known about the interfaces that govern binding between the subunits at the heart of the biomedically relevant telomerase RNP enzyme. Here, we have demonstrated that the core-enclosing helix of telomerase RNA is essential for binding to the TERT catalytic protein subunit in yeast.

In order to study the core-enclosing helix, which is in the middle of the area of required connectivity, we moved the ends of telomerase RNA away from the ARC so as not to disrupt it in our CEH mutant alleles. Thus, we circularly permuted TLC1 RNA, allowing us to delete the CEH without disrupting RNA continuity through the ARC. This approach afforded us the ability to parse ARC from CEH function, revealing that the CEH is essential for telomerase RNP function both *in vivo* and *in vitro*, consistent with it being required for binding to TERT. Furthermore, our findings are based on data obtained from studying the CEH within two independent circularly permuted telomerase RNA allele contexts (Fig. 2 and 3). One of the circular permutations has the RNA ends moved to the distal portion of the template-boundary element, cpTBE, and the other to within the junction in the central hub between the template and pseudoknot, cpJ3 (Fig. 1A). Under both of these different conditions, we found that a 4-bp CEH is sufficient for telomerase function. The presence of fewer base pairs did not permit CEH function, and it is likely that they also do not stably form a helix, consistent with a paired secondary structure element being necessary at this position of yeast telomerase RNA for it to function.

As for any functional importance of the sequence of the CEH, our results from sequence-randomized, truncated, and extended-helix alleles provide strong evidence that core-enclosing helices with diverse sequences (6 different variations) provide at least basic telomerase activity. Functionality of the CEH in these alleles was despite the sensitized context of being tested within miniaturized TLC1 RNA, which, even when otherwise wild type in sequence, supports rather short telomeres. We conclude from the results that the sequence of the CEH, although making some degree of contribution to telomerase RNA function, is not essential and therefore that the CEH secondary structure is more important than the primary structure.

A core-enclosing helix is a conserved feature of telomerase RNAs beyond yeasts, being also present in human, ciliate, and most other telomerase RNAs known to date. The 4 bp that we find to be critical in the S. cerevisiae CEH are essentially invariant among *Saccharomyces* species (5, 14), with the most core-proximal G-C changing to a G·U pair in one strain of Saccharomyces mikatae (8), consistent with our results suggesting that secondary structure is more important than sequence. In the ciliated protozoan Tetrahymena thermophila, disruption of the core-enclosing helix reduces activity by 95 to 99%, indicating the importance of this structure in a distantly related species (15). In humans, while truncating the 5′ end still permits functional telomerase, template-boundary definition is compromised (16). Curiously, rodent telomerase RNAs have their 5′ end located just 2 to 5 nucleotides upstream of the template (17, 18), and therefore, presumably, these nucleotides cannot pair to form a CEH. In these species, it has been proposed that TERT simply uses runoff reverse transcription, since these RNAs also lack a template-boundary element (17). Thus, rodent RNAs differ in this way from those of all other known telomerases. It is worth also noting that the template-boundary element and core-enclosing helix appear to be consolidated into a single paired element in human telomerase RNA (11, 16). Accordingly, the CEH in yeast could be an essential TERT-binding site while not performing the orthologous function in some other species. It is likely that the differences with respect to how the RNA and TERT interact are due to the rapid evolution of the RNP.

Our results show that binding of TERT to TLC1 does not strictly require a specific helix sequence but rather appears to be dictated more by secondary structure. This conclusion is supported by phylogenetics and the fact that the 6 different paired sequences that we tested in place of the wild-type CEH provided the essential function *in vivo* and *in vitro* (Fig. 2 to 4). The CARRY two-hybrid assay was not able to detect binding to TERT for the random 4-bp CEH and detected weak binding for the 23-nt CEH (Fig. 5B). This suggests that the TERT-binding affinity for the random and 23-nt CEH RNAs is around the threshold for the CARRY two-hybrid assay and is consistent with the reduced *in vitro* telomerase activity supported by these alleles compared to 4-bp CEH (Fig. 2D, lane 9, and Fig. 3D, lanes 8 and 10). Overall, our data suggest that TERT binds directly to the core-enclosing helix of yeast telomerase RNA. Nevertheless, it is feasible that the CEH also, or instead, promotes binding of TERT elsewhere on the RNA. It is quite likely that the CEH is not the only binding site for TERT in TLC1, particularly given the complex dynamics required in telomerase for iterative template reuse during repeat addition processivity. As for which of the other conserved secondary structure elements in yeast telomerase RNA might also be making binding contacts with TERT, some of the best possibilities include the pseudoknot, template-boundary element (TBE), and/or core junctions (J1, J2, and J4). A previous study showed that truncations of the pseudoknot lead to reduced binding of TERT to Micro-T but that the triple-helix region was not involved in binding (9). Although the simplest way to explain these results is that TERT binds to the pseudoknot, it is possible that the tested pseudoknot truncations have also destabilized the CEH, which is located just 3′ of several of the most binding-defective pseudoknot alleles. With respect to TERT binding to core junctions, we previously showed that junction 1 is dispensable for basal function but the 2-nt junction 4, between the CEH and the pseudoknot (Fig. 1A), is essential for telomerase activity in the ARC (8).

A 3-Å crystal structure of the *Tetrahymena* TERT RNA-binding domain in complex with the TBE helix shows that the protein makes direct contacts with the unpaired nucleotides at the base of this helix (19). The 4.8-Å cryo-electron microscopy (cryo-EM) structure of the *Tetrahymena* holoenzyme (20) shows that TERT contacts the RNA around the periphery of the core enzyme RNP complex, where there is space that could allow multiple TERT-binding interfaces in the evolutionarily distant yeast species and perhaps even also in ciliates. Integration of the yeast and ciliate results suggests that yeast TERT may also bind to CEH-flanking junctions in TLC1. However, J1 contacts are not absolutely required for telomerase function, since this junction is not essential for basal activity (8). In terms of the human telomerase 10.2-Å cryo-EM structure (21), the P1b helix (i.e., the “CEH” in human telomerase RNA, hTR) is juxtaposed to the modeled *Tribolium* RNA-binding domain of TERT, so it very well could represent a key binding interface for hTERT in the human core enzyme as well, especially when considering the ciliate TERT-RBD binding to the ciliate TBE helix.

**FIG 6:**
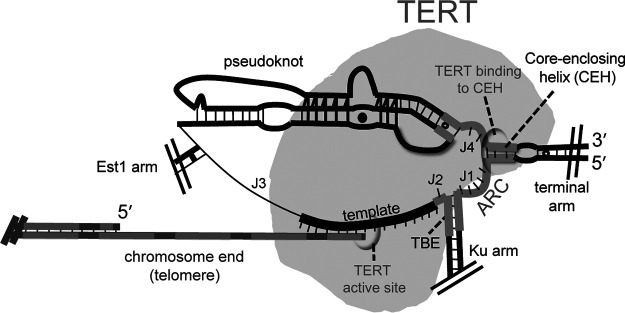
RNA-TERT interactions and their functional implications for telomere extension by the yeast telomerase RNP core enzyme. The core-enclosing helix and its binding to TERT (gray) are indicated. We hypothesize that binding of TERT to the region(s) connected to the template by the area of required connectivity (dark gray) allows TERT to stay associated with TLC1. This binding interaction is likely to be important during the unique telomerase reverse transcription catalytic cycle that is based on an intrinsic RNA template, which requires the TERT active site to dissociate from the template and newly synthesized DNA during repeat addition processivity *in vivo*.

Telomerase is a unique reverse transcriptase in that it, unlike viral reverse transcriptases, iteratively reuses a short RNA template intrinsic to the RNP enzyme. This template reuse, required for processive activity, involves transient disruption of the base pairing of the RNA with the enzyme’s DNA substrate, while the RNP remains associated with the chromosome end via another telomere-enzyme binding interaction(s). During this multifaceted interaction of the enzyme with telomeric DNA through the catalytic cycle, the telomerase RNA-TERT interaction must also be maintained. This dynamic interplay should necessitate a multipartite binding interaction between the telomerase RNA and TERT. Telomerase, of course, interacts with the template via its active site, but presumably the highest-affinity binding interaction between the RNA and TERT resides outside the RNA’s template region in order to provide a connection that maintains core RNP integrity during catalysis. It may be important that this TERT-binding site in the RNA is distal to the TERT catalytic site-RNA template part of the RNP so as to avoid the binding site sterically hindering the repeated process of binding, extending, and dissociating from the telomeric DNA substrate. Specifically, as discussed above, we propose that TERT binds most prominently to telomerase RNA nucleotides elsewhere around the RNA’s central hub from the template in the secondary structure model (Fig. 6). This is supported by our current results and those published from various labs studying yeast and other organisms (9, 11, 19, 20, 22), including our prior finding that breaking the RNA backbone in the area of required connectivity (ARC), which connects the core-enclosing helix to the template, abolishes telomerase core enzyme activity (8). In summary, as summarized by Fig. 6 for the case of yeast, we propose that there is a central theme emerging among telomerases: a critical helix in the hub of telomerase RNAs comprises an essential TERT-binding foothold that keeps the RNP core enzyme lashed together during rounds of RNA template-directed DNA synthesis.

## MATERIALS AND METHODS

### Yeast strains and plasmids.

All *in vivo* assays for telomerase function used yeast strain yVL009 (*MAT***a** *tlc1*Δ::*LEU2 rad52*-Δ::*LYS ura3-52 lys2-801 ade2-101 trp-1*-Δ*1 his3*-Δ*200 leu2*-Δ *cir^+^* pSD120/p*TLC1-URA3-CEN*) (22). All new mutants were made by PCR and subcloned into a 2μ *TRP1*-marked pRS424 vector for *in vivo* assays or a pUC19-based vector for *in vitro* assays.

### Senescence assays.

yVL1009 yeast strains were transformed with a *TRP1*-marked *CEN* or 2μ plasmid via standard lithium acetate methods. Transformants were selected on medium lacking uracil and tryptophan and then streaked on medium lacking tryptophan and containing 5-fluoroorotic acid (5-FOA) to counterselect for the wild-type *URA3*-marked TLC1 cover plasmid. Duplicate colonies of yeast containing only the transformed plasmid were then serially streaked 10 times on medium lacking tryptophan at 30°C.

### Southern blots.

To determine telomere length, Southern blots were performed as previously described (6). Briefly, yeast cells were cultured in liquid medium lacking tryptophan at 30°C, and genomic DNA was isolated (Puregene kit; Qiagen). Purified genomic DNA was digested overnight with XhoI and then electrophoresed through a 1.1% agarose gel at 70 V for 17 h. DNA fragments were then transferred to a Hybond N^+^ membrane (GE Healthcare) by capillary action, cross-linked with UV irradiation, and incubated with radiolabeled probes for telomeric DNA and a region of chromosome IV. Blots were imaged using a Typhoon 9410 variable mode imager.

### Northern blots.

Total RNA was isolated, using a hot phenol extraction method, from log-phase yeast liquid cultures grown in the absence of tryptophan. Thirty micrograms of total cellular RNA was electrophoresed through a 4% polyacrylamide–1× Tris-borate-EDTA–7 M urea gel at 35 W for ∼3 h. Separated RNAs were electrotransferred to a Hybond N^+^ membrane (GE Healthcare) and UV cross-linked. The membrane was probed with 1 × 10^7^ cpm of an StuI-NsiI fragment of Mini-T(460) and 1 × 10^5^ cpm of the control RNA, *NMEI*. Blots were imaged using a Typhoon 9410 variable mode imager.

### Reconstituted telomerase activity assays.

Telomerase was made using a coupled *in vitro* transcription and translation system as previously described (3). Briefly, DNA templates for ProA-Est2 TERT and telomerase RNA were incubated in a rabbit reticulocyte lysate system, including [^35^S]methionine, for 90 min. Telomerase was then immunopurified using IgG-Sepharose beads (GE Healthcare). Purified telomerase was incubated with telomeric DNA oligonucleotide (DZ428: 5′-GGTGTGGTGTGGG-3′), a 5′ γ-^32^P-labeled internal recovery and loading control, [α-^32^P]dTTP, and 1 mM each unlabeled dATP, dCTP, and dGTP in standard *in vitro* telomerase activity buffer. Assembled reaction mixtures were incubated for 10 min at 26°C, and then reactions were stopped by the addition of ammonium acetate and ethanol precipitated. Products were resuspended in formamide loading dye and boiled at 95°C for 5 min. Samples were electrophoresed through a 10% polyacrylamide–1× Tris-borate-EDTA–7 M urea gel for 75 min at 90 W and then dried on Whatman paper and exposed to a phosphorimager screen. Imaging was done with a Typhoon 9410 variable mode imager. As a loading and recovery control during purification of reconstituted telomerase (Fig. 2D, 3D, and 4E), 2.5 ml of telomerase was resuspended in Laemmli sample buffer, boiled at 95°C for 5 min, and separated on a 7.5% Mini-PROTEAN TGX gel (Bio-Rad). The gel was transferred to Whatman paper, dried, and imaged using a Typhoon 9410 variable mode imager.

### CARRY two-hybrid RNA-RBP assays.

CARRY two-hybrid assays were carried out as described previously (13). Briefly, the strain CARRYeast-1a [*MAT***a** *his3*Δ*200 trp1-901 ade2 lys2*::(*4LexAop-HIS3*) *ura3*::(*8LexAop-LacZ*) *leu2*::(*KanMX6_dCas9*)] was transformed with single guide RNA (sgRNA) fusion and GAD fusion plasmids with the vector backbones from *TRP1*-marked pCARRY2 (13) and *LEU2*-marked pGAD424 (23), respectively. Transformants were grown to saturation in synthetic liquid culture medium lacking tryptophan and leucine. Six 10-fold serial dilutions were made of each culture, and 5 μl of each dilution, as well as the undiluted culture, was pipetted onto both solid –Trp –Leu and –Trp –Leu –His minimal media. Cells were incubated for 3 days at 30°C and photographed.
